# The association of coronary artery disease with heart rate at anaerobic threshold and respiratory compensatory point

**DOI:** 10.3389/fcvm.2024.1442857

**Published:** 2024-10-02

**Authors:** Yiya Kong, Ruihuan Shen, Tao Xu, Jihong Zhou, Chenxi Xia, Tong Zou, Fang Wang

**Affiliations:** ^1^ Department of Cardiology, Beijing Hospital, National Center of Gerontology, Institute of Geriatric Medicine, Chinese Academy of Medical Sciences, Beijing, China; ^2^ Graduate School of Peking Union Medical College, Chinese Academy of Medical Sciences, Beijing, China

**Keywords:** heart rate, coronary artery disease, anaerobic threshold, respiratory compensatory point, cardiopulmonary exercise test

## Abstract

**Background:**

There is limited knowledge regarding the association between heart rate (HR) during different exercise phases and coronary artery disease (CAD). This study aimed to evaluate the relationship between four exercise-related HR metrics detected by cardiopulmonary exercise testing (CPET) and CAD. These metrics include HR at the anaerobic threshold (HR_AT_), HR at respiratory compensatory point (HR_RCP_), maximal HR (HR_max_), and HR 60 s post-exercise (HR_Rec60s_).

**Methods:**

The 705 participants included 383 with CAD and 322 without CAD in Beijing Hospital, who underwent CPET between January 2021 and December 2022. The Logistic regression analysis was applied to estimate the odds ratio and the 95% confidence interval. Additionally, the multivariable Logistic regression analyses with restricted cubic splines were conducted to characterize the dose-response association and explore whether the relationship was linear or nonlinear.

**Results:**

Our primary finding indicates that for each one-beat increase in HR_AT_, there is a 2.8% reduction in the adjusted risk of CAD in the general population. Similarly, a one-beat increase in HR_RCP_ corresponds to a 2.6% reduction in the adjusted risk of CAD. Subgroup analyses revealed significant interactions between HR_AT_ and factors such as sex, hypertension, and lung cancer, as well as between HR_RCP_ and sex and hypertension, in relation to CAD. The dose-response analysis further confirmed that higher HR_AT_ and HR_RCP_ are associated with a reduced risk of CAD.

**Conclusion:**

These results are suggestive of a good association between HR_AT_, HR_RCP_, and CAD. The lower HR_AT_, and HR_RCP_ are signs of poor HR response to exercise in CAD. HR_AT_ and HR_RCP_ are potentially good indicators of poor HR response to exercise without considering maximal effort.

## 1 Introduction

Coronary artery disease (CAD) is a leading cause of morbidity and mortality, posing a growing public health burden worldwide (1). Prediction and early diagnosis of CAD facilitates appropriate intervention in its early stages, aiming to improve the prognosis, delay the progression, and reduce the burden on patients and their families. In accordance with the latest guidelines from the American College of Cardiology and the American Heart Association, exercise stress testing was recommended as an initial diagnostic test for suspected CAD patients (2).

Cardiopulmonary exercise testing (CPET) is a non-invasive method of evaluating an individual's cardiovascular, muscular, respiratory, and metabolic responses to physical stress through exercise stress testing combined with expired gas analysis (3). Unlike the current “gold standard” for diagnosing CAD, which relies on invasive coronary angiography, CPET offers a safer, less expensive, and more psychologically comfortable alternative for patients. Although CPET involves a variety of complex indicators, each parameter provides distinct diagnostic and prognostic insights, making this area increasingly important in clinical practice.

We will emphasize the two vital lactic acid (LA)-related metabolism points during exercise in our study. As the incremental exercise testing proceeds, the LA in the circulation begins to accumulate eventually leading to hypercapnia at the end of the exercise (4). Once a working skeletal muscle cell begins to produce LA, the anaerobic threshold (AT) is reached. AT marks the transition to mixed aerobic-anaerobic metabolism (5). The work intensity increases continuously and gradually to go beyond a certain point called the respiratory compensation point (RCP), where LA production can no longer be compensated by circulating bicarbonate, then hyperventilation begins. RCP represents the transition to predominant anaerobic metabolism (5).

Heart rate (HR) is thought to have a broad and complex relationship with the cardiac function of CAD. However, the significance of HR, particularly exercise-induced HR, in understanding cardiovascular pathophysiology, prognosis, and treatment is often underestimated. This may be due to the complex nature of its effects, despite HR being a familiar and easily measurable parameter (6). Previous evidence has consistently shown that elevated resting heart rate (HR) is an independent predictor of both all-cause and cardiovascular mortality in patients with CAD (7–9). Additionally, poor exercise capacity and inadequate HR response during exercise and recovery are significant indicators of higher overall mortality and an increased risk of CAD (10–12). However, most previous studies have primarily focused on resting HR and HR recovery post-exercise in CAD patients, often neglecting the importance of HR at various stages of exercise.

In this study, we examined four specific HR metrics during different phases of CPET as potential predictors of CAD: HR at the anaerobic threshold (HR_AT_), HR at the respiratory compensatory point (HR_RCP_), maximal HR (HR_max_), and HR 60 s post-exercise (HR_Rec60s_). This cross-sectional, population-based study aimed to compare these exercise-related HR measurements to determine which one is most strongly associated with CAD in the general population.

## 2 Method

### 2.1 Ethics statements

This cross-sectional study conformed to the Declaration of Helsinki and was approved by the Committee of Beijing Hospital (2023BJYYEC-116-01).

### 2.2 Study population

This cross-sectional study included 705 patients, aged 18–60, who underwent CPET at Beijing Hospital between January 2021 and December 2022. The testing was conducted to screen for cardiopulmonary disease or to evaluate exercise capacity and/or the severity of CAD.

The diagnosis of coronary artery disease (CAD) was confirmed by reviewing each patient's inpatient and/or outpatient medical records. Documented CAD was defined by the presence of at least one of the following criteria: (1) ≥50% stenosis in at least one coronary artery trunk or major branch as demonstrated by percutaneous coronary angiography or computed tomography; (2) typical exertional angina symptoms with a positive stress test (electrocardiogram stress test, stress echocardiography, or nuclear myocardial stress imaging); (3) previously diagnosed myocardial infarction; (4) previously diagnosed unstable angina pectoris (typical ischemic chest pain + ECG changes + increased markers of muscle damage; or the dynamic changes of ST segment during ischemic attack, or coronary angiography confirmed the existence of severe lesions leading to symptoms) (13, 14). According to the history of CAD, there were 322 participants in the non-CAD group and 383 participants in the CAD group.

### 2.3 Data collection

Baseline characteristics for the target population were gathered from electronic medical records. These included demographics, comorbidities, chronic medications, past medical history, and biochemical data such as total cholesterol (TC), triglyceride (TG), high-density lipoprotein cholesterol (HDL-C), low-density lipoprotein cholesterol (LDL-C), and N-terminal pro-B-type natriuretic peptide (NT-proBNP) or brain natriuretic peptide (BNP).

### 2.4 Cardiopulmonary exercise test

The cardiopulmonary function detector (MasterScreen CPX, Jaeger, Switzerland) was used to detect the changes of oxygen consumption (VO_2_) and carbon dioxide (VCO_2_) emission on an upright cycle ergometer (Ergoselect 100p, Ergoline, Germany) or mechanical treadmill (T2100-ST2, GE, America). Meanwhile, a 12-lead ECG recorder (CASE, GE, America) and a dynamic blood pressure monitor (TangoM2, SunTech, America) records continuously the HR and blood pressure (BP).

CPET monitored the following parameters for each participant at resting, AT, RCP, and peak states, and 1, 2 and 3 min after exercise, including work load (WL), minute ventilation (VE), VO_2_, oxygen consumption/kilogram (VO_2_/kg; which is considered as the peak VO_2_ at the maximal WL), VCO_2_, HR, respiratory rate (RR), oxygen pulse (VO_2_/HR), dead space (VD), tidal volume (VT), systolic blood pressure (SBP), diastolic blood pressure (DBP), breathing reserve (BR), respiratory quotient (RQ), end-tidal carbon dioxide pressure (P_ET_CO_2_), end-tidal oxygen pressure (P_ET_O_2_), and oxygen saturation (SpO_2_). The VE/VCO_2_ was calculated. The detailed CPET assessment protocol includes a 3-min rest and 3-min warm-up at 0 watts (W), followed by a continuous increase in the Work Rate (WR) by 10 W/min to 20 W/min until exhaustion. A respiratory exchange ratio (RER, ratio of VCO_2_/VO_2_ at peak exercise) of ≥1.05 was considered an objective indicator of peak effort during assessment (15). Borg Scale (scale 6–20) > 17 was regarded as a subjective index. Discontinue the exercise test if any of the following occurs: abnormal hemodynamic or ECG exercise response or other reasons such as dyspnea, angina, or lower extremity muscle fatigue (16, 17).

AT and RCP were located by visual inspection. AT is deemed reached when the following criteria are met: (1) the VE/VO_2_ curve starts to rise with the VE/VCO_2_ curve remaining constant, and (2) P_ET_O_2_ starts to rise with P_ET_CO_2_ remaining unchanged. RCP is deemed to be reached when the following criteria are met: (1) a decrease in P_ET_CO_2_ after reaching a maximal level; (2) a rapid nonlinear increase in VE (second deflection); (3) the VE/VCO_2_ ratio reached a minimum and began to increase and (4) a nonlinear increase in VCO_2_ vs. VO_2_ (departure from linearity) (4, 18).

### 2.5 Heart rate

Continuous ambulatory electrocardiograms were recorded using a 12-lead ECG recorder (CASE, GE, America) during the maximum symptom-limited CPET. HR was recorded at AT and RCP, designated as HR_AT_ and HR_RCP_, respectively. HR_max_ was defined as the highest HR achieved during the CPET. Additionally, HR 60 s after the exercise session, referred to as HR_Rec60s_, was measured during the recovery period.

### 2.6 Statistical analysis

The random forest method was used to impute missing data (19). Based on their CAD history, participants were categorized into a CAD group and a non-CAD group. The distributions of variables in each group were assessed using the Kolmogorov-Smirnov test. Continuous variables that followed a normal distribution were reported as mean ± standard deviation and analyzed using Student's *t*-test. For variables that did not follow a normal distribution, data were presented as median and interquartile range (IQR), with the rank-sum test applied for comparison. Categorical variables were expressed as counts and percentages and compared using the Chi-square test.

Logistic regression analysis was used to estimate the odds ratios (OR) and 95% confidence intervals (CIs) for the continuous HR-related indices (HR_RCP_, HR_AT_, HR_max_, and HR_Rec60s_) in relation to the outcome of CAD. Additionally, we categorized the HR-related indices into tertiles and compared the associations between the medium and highest tertiles with the lowest tertile. Subgroup analysis was performed to determine whether the relationship between CAD and HR-related indices differed across various subgroups defined by covariates and comorbid conditions. We examined the interaction effects of CAD with HR-related indices in several participant subgroups (age grouping, sex, hypertension, diabetes mellitus, hyperlipidemia, and lung cancer status), and the Wald test determined the *P* for interaction. We constructed three multivariable Logistic regression models. Model 1 was unadjusted. The second and third adjustment models with robust adjustment for covariates are thought to be potential confounders of the associations of the HR-related indices with CAD. Thus, model 2 included age (Continuous), sex (male or female), and body mass index [normal (18.5–25 kg/m^2^), overweight (≥25 kg/m^2^) or low (<18.5 kg/m^2^)]; model 3 further adjusted for hypertension, diabetes mellitus, hyperlipidemia, chronic kidney disease, and lung cancer status (yes or no).

The multivariable Logistic regression analyses with restricted cubic splines (RCS) were used to characterize the dose-response association and explore the potential linear or nonlinear relationship of the HR-related indices with the CAD. The Akaike information criterion (AIC) was used to identify the knots for the splines to balance best fit and overfitting in the RCS (20). The medians of the HR-related indices were assigned as the reference values. The test result for nonlinearity was checked first. If the test for nonlinearity was insignificant, the overall association test result was checked, with the considerable result indicating the linear association.

For statistical analysis, R (version 4.2.2; https://www.R-project.org) was utilized. A result with a two-sided *P* value < 0.05 was considered statistically significant when testing the hypotheses of the study.

## 3 Result

### 3.1 Comparison of baseline characteristics between CAD group and non-CAD group

A total of 705 eligible participants met all the inclusion and none of the exclusion criteria. The average age was 59.40 ± 11.44 years, and 408 (57.90%) were men. Compared with the non-CAD group, participants with CAD tended to be male, older, and have higher BMI, VO_2_/HR in the period of resting, AT, RCP, and peak (all *P* < 0.05). The proportions of Co-morbid conditions, including hypertension, diabetes mellitus (DM), hyperlipidemia, chronic kidney disease (CKD), and stroke were higher in the CAD group. The participants in the non-CAD group had higher TC, HDL-C, LDL-C, and HR in the period of resting, AT, RCP, and peak, and VO_2_/kg in the period of AT, RCP, and peak, and lower percentage of lung cancer, than in the CAD group (all *P* < 0.05) (Table 1). The baseline characteristics of participants grouped by HR-related indices tertiles were shown in the Supplementary Tables S1–S4.

**Table 1:** Baseline characteristics of 705 participants.

	non-CAD (*n* = 322)	CAD (*n* = 383)	*P*
Sex (%)			<0.0001
Female	164 (50.93)	133 (34.73)	
Male	158 (49.07)	250 (65.27)	
Age (year), mean (SD)	56.957 (12.534)	61.462 (9.987)	<0.0001
BMI, median (IQR)	24.340 (22.389, 26.943)	25.391 (23.405, 27.470)	0.0003
BMI (%)			0.0001
Normal	177 (54.97)	176 (45.95)	
Overweight	134 (41.61)	206 (53.79)	
Low	11 (3.42)	1 (0.26)	
Hypertension (%)	161 (50.00)	312 (81.46)	<0.0001
DM (%)	61 (18.94)	144 (37.60)	<0.0002
Hyperlipidemia (%)	126 (39.13)	332 (86.68)	<0.0004
CKD (%)	9 (2.80)	26 (6.79)	0.024
Lung cancer (%)	104 (32.30)	41 (10.70)	<0.0002
OSA (%)	14 (4.35)	24 (6.27)	0.339
COPD (%)	10 (3.11)	11 (2.87)	1
Stroke (%)	303 (94.10)	337 (87.99)	0.0078
Serum indexes			
NT-proBNP (pg/ml), mean (SD)	249.071 (590.978)	251.864 (792.218)	0.9584
BNP (pg/ml), mean (SD)	69.653 (62.369)	80.849 (124.174)	0.1421
TC (mmol/L), mean (SD)	4.606 (0.888)	4.148 (0.989)	<0.0001
TG (mmol/L), mean (SD)	1.574 (1.260)	1.542 (1.043)	0.7128
HDL-C (mmol/L), mean (SD)	1.226 (0.281)	1.155 (0.305)	0.0015
LDL-C (mmol/L), mean (SD)	2.779 (0.762)	2.400 (0.889)	<0.0001
Resting			
VO_2_ (L/min), median (IQR)	309.500 (259.000, 378.000)	320.000 (265.000, 383.500)	0.2818
VO_2_/kg (ml/kg/min), median (IQR)	4.600 (3.900, 5.575)	4.500 (3.800, 5.200)	0.068
HR (bpm), median (IQR)	81.000 (73.250, 88.000)	75.000 (67.000, 83.000)	<0.0001
VO_2_/HR (ml/beat), median (IQR)	3.900 (3.200, 4.900)	4.400 (3.500, 5.300)	0.0001
AT			
VO_2_ (L/min), median (IQR)	827.000 (679.500, 1,082.250)	837.000 (671.500, 1,061.000)	0.9156
VO_2_/kg (ml/kg/min), median (IQR)	12.350 (10.400, 14.800)	11.500 (9.500, 14.700)	0.0033
HR (bpm), median (IQR)	108.000 (98.000, 118.000)	99.000 (88.500, 108.500)	<0.0001
VO_2_/HR (ml/beat), median (IQR)	7.900 (6.300, 9.850)	8.600 (7.000, 10.600)	0.0007
ΔVO_2_/ΔWR (ml/min/Watt), median (IQR)	9.549 (8.032, 11.178)	9.920 (8.450, 11.815)	0.1095
Peak			
VO_2_ (L/min), median (IQR)	1,291.500 (1,038.500, 1,618.000)	1,323.000 (1,052.000, 1,601.000)	0.9904
VO_2_/kg (ml/kg/min), median (IQR)	19.200 (16.225, 22.875)	17.700 (15.000, 22.200)	0.0039
HR (bpm), median (IQR)	137.000 (123.500, 153.000)	125.000 (112.000, 141.000)	<0.0001
VO_2_/HR (ml/beat), median (IQR)	9.400 (7.800, 12.075)	10.600 (8.500, 12.650)	0.0001
ΔVO_2_/ΔWR (ml/min/Watt), median (IQR)	9.785 (8.400, 11.035)	9.700 (8.435, 11.080)	0.9882
RCP			
VO_2_ (L/min), median (IQR)	1,067.000 (859.250, 1,367.633)	1,090.000 (873.500, 1,354.000)	0.7092
VO_2_/kg (ml/kg/min), median (IQR)	15.850 (13.404, 19.100)	15.100 (12.700, 18.533)	0.0144
HR (bpm), median (IQR)	126.000 (114.000, 139.000)	113.000 (101.000, 127.000)	<0.0001
VO_2_/HR (ml/beat), median (IQR)	8.500 (7.100, 10.975)	9.600 (7.881, 11.600)	0.0001
ΔVO_2_/ΔWR (ml/min/Watt), median (IQR)	9.390 (8.110, 10.508)	9.330 (8.305, 10.698)	0.4039
Rec60 s			
VO_2_ (L/min), median (IQR)	620.000 (506.250, 751.500)	653.000 (551.000, 789.500)	0.007
VO_2_/kg (ml/kg/min), median (IQR)	11.900 (10.300, 13.900)	11.700 (10.200, 13.800)	0.731
HR (bpm), median (IQR)	119.000 (107.000, 136.000)	109.000 (96.500, 124.000)	<0.0001
VO_2_/HR (ml/beat), median (IQR)	6.700 (5.600, 8.400)	7.900 (6.400, 9.550)	<0.0001
ΔVO_2_/ΔWR (ml/min/Watt), median (IQR)	36.085 (27.340, 45.245)	36.620 (28.860, 49.695)	0.1231

BMI, body mass index; DM, diabetes mellitus; CKD, chronic kidney disease; OSA, obstructive sleep apnea; COPD, chronic obstructive pulmonary disease; BNP, brain natriuretic peptide; TC, total cholesterol; TG, triglyceride; VO_2_, oxygen consumption; VO_2_/kg, oxygen consumption/kilogram; HR, heart rate; VO_2_/HR, oxygen pulse; ΔVO_2_/ΔWR, ratio of the increase in VO_2_ to the increase in the work rate; AT, anaerobic threshold; RCP, respiratory compensation point; Rec60 s, post-exercise after 60 s; SD, standard deviation; IQR, interquartile range.

### 3.2 Multivariable logistic regression analysis of HR-related indices associated with CAD

Tables 2, 3 shows the results of associations between HR-related indices and risk of CAD using the multivariable Logistic regression analysis. The fully multivariable-adjusted ORs (95% CIs) per 1 unit increase of HR_AT_, HR_RCP_, HR_max_, and HR_Rec60s_ for CAD were 0.972 (0.960, 0.984), 0.974 (0.963, 0.985), 0.980 (0.971, 0.990), and 0.984 (0.975, 0.993), respectively. Compared with participants with HR_AT_ <96 bpm, the multivariable-adjusted ORs (95% CIs) were 0.618 (0.390, 0.974) and 0.368 (0.229, 0.589) for CAD in participants with HR_AT_ ranged from 96 to 110 bpm and ≥110 bpm. Compared with participants with HR_RCP_ < 111 beats per minute (bpm), the multivariable-adjusted ORs (odds ratio, 95% CIs) were 0.394 (0.244, 0.629) and 0.336 (0.204, 0.546) for CAD in participants with HR_RCP_ ranged from 111 to 127 bpm and ≥127 bpm. Compared with participants with HR_max_ < 121 bpm, the multivariable-adjusted ORs (95% CIs) were 0.448 (0.279, 0.713) and 0.317 (0.192, 0.518) for CAD in participants with HR_max_ ranged from 121 to 142 bpm and ≥142 bpm. Compared with participants with HR_Rec60s_ < 105 bpm, the multivariable-adjusted ORs (95% CIs) were 0.375 (0.232, 0.597) and 0.376 (0.231, 0.608) for CAD in participants with HR_Rec60 s_ ranged from 105 to 123 bpm, and ≥123 bpm.

**Table 2:** Multivariable logistic regression analysis of HR_AT_ and HR_RCP_ associated with CAD.

HR_AT_						HR_RCP_		
	Continuous	< 96	[96,110)	≥110	Continuous	<111	[111, 127)	≥127
Model 1								
OR	0.963 (0.953,0.973)	1.000 (R.)	0.516 (0.351,0.755)	0.248 (0.168,0.364)	0.966 (0.958,0.975)	1.000 (R.)	0.422 (0.286,0.619)	0.254 (0.172,0.373)
*P* values	<0.0001		<0.001	<0.0001	<0.0001		<0.0001	<0.0001
Model 2								
OR	0.971 (0.960,0.981)	1.000 (R.)	0.593 (0.399,0.878)	0.336 (0.223,0.503)	0.973 (0.964,0.982)	1.000 (R.)	0.482 (0.323,0.715)	0.350 (0.231,0.529)
*P* values	<0.0001		0.009	<0.0001	<0.0001		<0.001	<0.0001
Model 3								
OR	0.972 (0.960,0.984)	1.000 (R.)	0.618 (0.390,0.974)	0.368 (0.229,0.589)	0.974 (0.963,0.985)	1.000 (R.)	0.394 (0.244, 0.629)	0.336 (0.204, 0.546)
*P* values	<0.0001		0.039	<0.0001	<0.0001		<0.001	<0.0001

**Table 3:** Multivariable logistic regression analysis of hR_max_ and HR_Rec60s_ associated with CAD.

	HR_max_				HR_Rec60s_			
	Continuous	< 121	[121,142)	≥ 142	Continuous	< 105	[105,123)	≥ 123
Model 1								
OR	0.974 (0.966,0.981)	1.000 (R.)	0.503 (0.342,0.737)	0.261 (0.176,0.383)	0.978 (0.971,0.986)	1.000 (R.)	0.417 (0.284,0.609)	0.314 (0.213,0.458)
*P* values	<0.0001		<0.001	<0.0001	<0.0001		<0.0001	<0.0001
Model 2								
OR	0.979 (0.971,0.987)	1.000 (R.)	0.525 (0.353,0.778)	0.346 (0.227,0.524)	0.984 (0.976,0.992)	1.000 (R.)	0.450 (0.302,0.667)	0.412 (0.273,0.618)
*P* values	<0.0001		0.001	<0.0001	<0.0001		<0.0001	<0.0001
Model 3								
OR	0.980 (0.971,0.990)	1.000 (R.)	0.448 (0.279, 0.713)	0.317 (0.192, 0.518)	0.984 (0.975,0.993)	1.000 (R.)	0.375 (0.232, 0.597)	0.376 (0.231, 0.608)
*P* values	<0.0001		<0.001	<0.0001	<0.001		<0.0001	<0.0001

Model 1: unadjusted model; Model 2: adjusted for age (Continuous), sex (male or female), and body mass index [normal (18.5–25 kg/m^2^), overweight (≥25 kg/m^2^) or low (<18.5 kg/m^2^)]; Model 3: Further adjusted for hypertension, diabetes mellitus, hyperlipidemia, chronic kidney disease, and lung cancer status (yes or no).

OR, odds ratio; R., reference; HR, heart rate; RCP, respiratory compensation point; AT, anaerobic threshold; max, maximum; Rec60 s, post-exercise after 60 s.

### 3.3 Subgroup analyses

Tables 4, 5 summarized the results of subgroup analysis between the HR-related indices and CAD according to different subgroups, including age, sex, hypertension, DM, hyperlipidemia, and lung cancer status, using multivariable Logistic regression analyses adjusting for age (continuous), sex (male or female), body mass index (BMI, normal [18.5–25 kg/m^2^], overweight [≥25 kg/m^2^] or low [<18.5 kg/m^2^], hypertension, diabetes mellitus, hyperlipidemia, chronic kidney disease, and lung cancer status (yes or no).

**Table 4:** Subgroup analysis of HR_RCP_ and HR_AT_ associated with CAD.

Characteristic	HR_AT_				HR_RCP_			
	<96	[96,110)	≥110	*P* for interaction	<111	[111, 127)	≥127	*P* for interaction
Age				0.467				0.527
<50years	1.000 (R.)	0.640 (0.148,2.774)	0.181 (0.039,0.834)		1.000 (R.)	0.266 (0.058,1.219)	0.291 (0.067,1.271)	
*P* value		0.551	0.028			0.088	0.101	
≥50years	1.000 (R.)	0.591 (0.362,0.965)	0.378 (0.227,0.629)		1.000 (R.)	0.384 (0.232,0.634)	0.284 (0.169,0.477)	
*P* value		0.035	<0.0001			<0.001	<0.0001	
Sex				0.001				0.001
Female	1.000 (R.)	0.434 (0.200,0.944)	0.152 (0.067,0.341)		1.000 (R.)	0.362 (0.161, 0.813)	0.167 (0.074, 0.380)	
*P* value		0.035	<0.0001			0.014	<0.0001	
Male	1.000 (R.)	0.707 (0.394,1.268)	0.623 (0.340,1.142)		1.000 (R.)	0.407 (0.225,0.737)	0.544 (0.288,1.025)	
*P* value		0.245	0.126			0.003	0.06	
Hypertension				0.036				0.021
Yes	1.000 (R.)	0.702 (0.405, 1.216)	0.314 (0.177, 0.555)		1.000 (R.)	0.349 (0.197, 0.619)	0.251 (0.138, 0.455)	
*P* value		0.207	<0.0001			<0.001	<0.0001	
No	1.000 (R.)	0.495 (0.201,1.215)	0.541 (0.227,1.288)		1.000 (R.)	0.625 (0.250,1.564)	0.746 (0.296,1.882)	
*P* value		0.125	0.165			0.316	0.535	
DM				0.475				0.52
Yes	1.000 (R.)	0.914 (0.376,2.223)	0.451 (0.197,1.029)		1.000 (R.)	0.572 (0.245,1.336)	0.441 (0.185,1.049)	
*P* value		0.843	0.059			0.197	0.064	
No	1.000 (R.)	0.543 (0.315,0.938)	0.338 (0.189,0.607)		1.000 (R.)	0.334 (0.187,0.598)	0.277 (0.150,0.509)	
*P* value		0.028	<0.001			<0.001	<0.0001	
Hyperlipidemia				0.104				0.718
Yes	1.000 (R.)	0.55 (0.315,0.961)	0.417 (0.230,0.757)		1.000 (R.)	0.376 (0.208,0.683)	0.322 (0.172,0.602)	
*P* value		0.036	0.004			0.001	<0.001	
No	1.000 (R.)	0.849 (0.376,1.918)	0.268 (0.111,0.651)		1.000 (R.)	0.462 (0.199,1.072)	0.342 (0.147,0.797)	
*P* value		0.694	0.004			0.072	0.013	
Lung Cancer				0.036				0.543
Yes	1.000 (R.)	1.639 (0.543,4.944)	0.357 (0.096,1.331)		1.000 (R.)	0.569 (0.190,1.708)	0.328 (0.104,1.036)	
*P* value		0.38	0.125			0.315	0.057	
No	1.000 (R.)	0.489 (0.290, 0.823)	0.342 (0.201, 0.581)		1.000 (R.)	0.373 (0.218, 0.637)	0.337 (0.193, 0.588)	
*P* value		0.007	<0.0001			<0.001	<0.001	

**Table 5:** Subgroup analysis of hR_max_ and HR_Rec60s_ associated with CAD.

Characteristic	HR_max_				HRRec60s			
	<121	[121,142)	≥142	*P*	<105	[105,123)	≥123	*P*
Age				0.417				0.572
<50years	1.000 (R.)	0.209 (0.040,1.083)	0.201 (0.045,.0.898)		1.000 (R.)	0.206 (0.037,1.159)	0.175 (0.034,0.911)	
*P* value		0.062	0.036			0.073	0.038	
≥50years	1.000 (R.)	0.461 (0.282,0.753)	0.28 (0.166,0.475)		1.000 (R.)	0.373 (0.227,0.612)	0.352 (0.212,0.587)	
*P* value		0.002	<0.0001			<0.0001	<0.0001	
Sex				0.027				0.134
Female	1.000 (R.)	0.421 (0.195, 0.913)	0.209 (0.095, 0.459)		1.000 (R.)	0.327 (0.153, 0.700)	0.304 (0.143, 0.646)	
*P* value		0.028	<0.0001			0.004	0.002	
Male	1.000 (R.)	0.459 (0.251,0.840)	0.405 (0.211,0.780)		1.000 (R.)	0.421 (0.228,0.777)	0.428 (0.225,0.813)	
*P* value		0.012	0.007			0.006	0.01	
Hypertension				0.037				0.054
Yes	1.000 (R.)	0.386 (0.219, 0.680)	0.251 (0.137, 0.458)		1.000 (R.)	0.336 (0.189, 0.595)	0.300 (0.166, 0.541)	
*P* value		0.001	<0.0001			<0.001	<0.0001	
No	1.000 (R.)	0.767 (0.303,1.945)	0.600 (0.239,1.509)		1.000 (R.)	0.575 (0.231,1.428)	0.678 (0.280,1.642)	
*P* value		0.577	0.278			0.233	0.389	
DM				0.928				0.684
Yes	1.000 (R.)	0.481 (0.202,1.141)	0.357 (0.151,0.843)		1.000 (R.)	0.538 (0.224,1.293)	0.435 (0.188,1.007)	
*P* value		0.097	0.019			0.166	0.052	
No	1.000 (R.)	0.400 (0.225,0.710)	0.283 (0.152,0.525)		1.000 (R.)	0.32 (0.179,0.570)	0.326 (0.177,0.601)	
*P* value		0.002	<0.0001			<0.001	<0.001	
Hyperlipidemia				0.276				0.656
Yes	1.000 (R.)	0.376 (0.208,0.679)	0.306 (0.163,0.574)		1.000 R.)	0.363 (0.203,0.649)	0.405 (0.219,0.748)	
*P* value		0.001	<0.001			<0.001	0.004	
No	1.000 (R.)	0.69 (0.310,1.538)	0.273 (0.110,0.679)		1.000 (R.)	0.401 (0.170,0.944)	0.346 (0.151,0.792)	
*P* value		0.364	0.005			0.037	0.012	
Lung cancer				0.329				0.462
Yes	1.000 (R.)	0.551 (0.204,1.485)	0.213 (0.056,0.805)		1.000 (R.)	0.45 (0.150,1.349)	0.278 (0.080,0.960)	
*P* value		0.238	0.023			0.154	0.043	
No	1.000 (R.)	0.438 (0.257, 0.748)	0.332 (0.191, 0.578)		1.000 (R.)	0.367 (0.215, 0.627)	0.392 (0.228, 0.675)	
*P* value		0.003	<0.0001			<0.001	<0.001	

R., reference; DM, diabetes mellitus; HR, heart rate; RCP, respiratory compensation point; AT, anaerobic threshold; max, maximum; Rec60 s, post-exercise after 60 s.

In subgroup analyses, statistically significant interactions were not observed between HR_Rec60s_ and any study covariates in relation to CAD (all *P* for interaction > 0.05). The interactions between HR_AT_ and sex, hypertension status, and lung cancer in relation to CAD were statistically significant (*P* for interaction = 0.001, 0.036, and 0.036, respectively). Statistically meaningful interactions were noted between HR_RCP_ and sex and hypertension status in relation to CAD (*P* for interaction = 0.001 and 0.021, respectively). The interactions between HR_max_ and sex and hypertension status in relation to CAD were also statistically significant (*P* for interaction = 0.027 and 0.037, respectively).

### 3.4 Dose-response analysis of the HR-related indices with CAD

**Figure 1:**
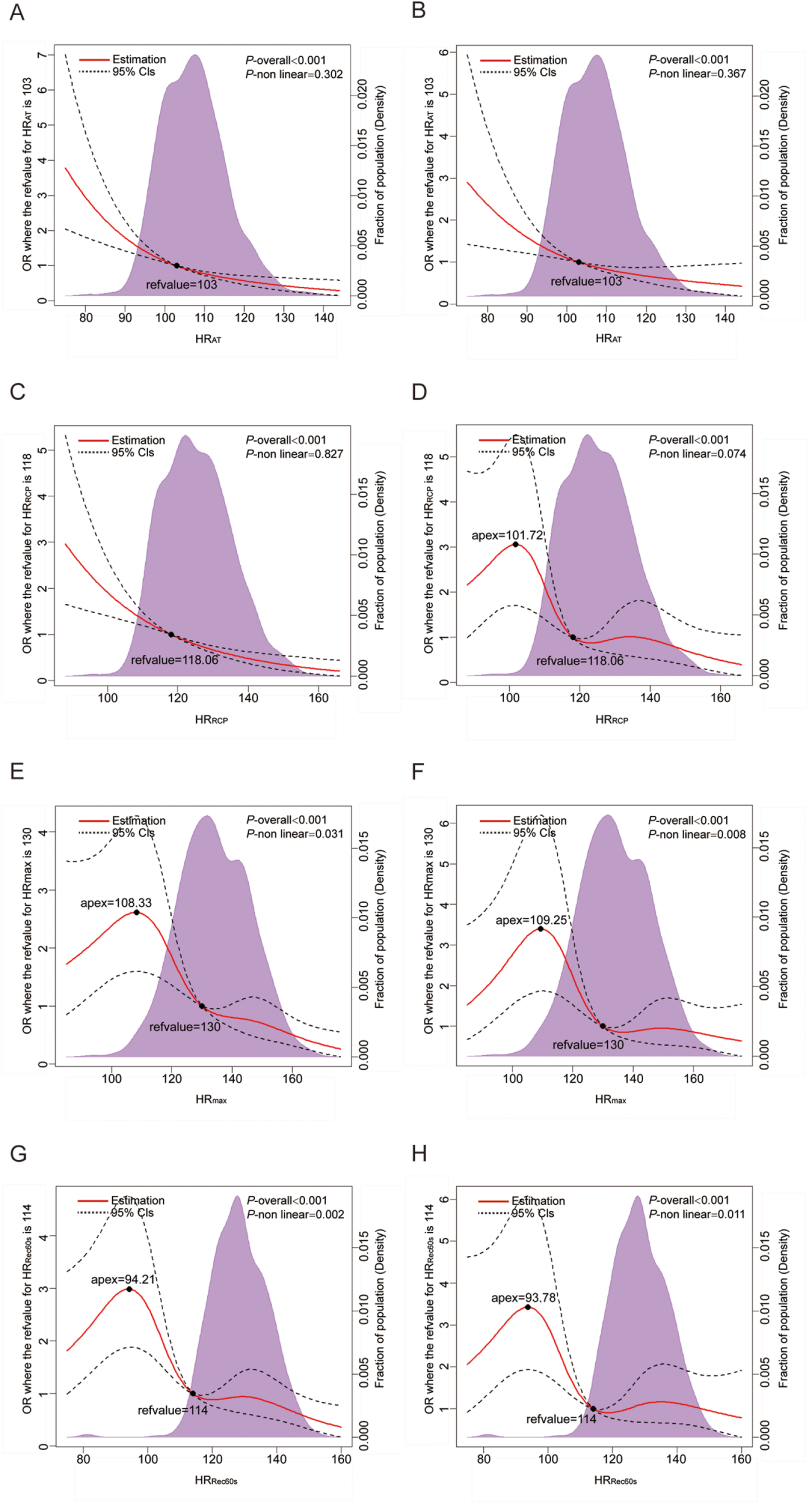
Dose–response analysis of the HR-related indices with CAD. **(A,C,E,G)** Dose–response analysis of HR_AT_, HR_RCP_, HR_max_, and HR_Rec60s_ using model 1; **(B,D,F,H)** Dose–response analysis of HR_AT_, HR_RCP_, HR_max_, and HR_Rec60s_ using model 3. Model 1: unadjusted model; Model 3: Further adjusted for hypertension, diabetes mellitus, hyperlipidemia, chronic kidney disease and lung cancer status (yes or no). HR, heart rate; RCP, respiratory compensation point; AT, anaerobic threshold; max, maximum; Rec60 s, post-exercise after 60 s.

Multivariable-adjusted RCS analyses revealed a linear association of HR_AT_ and HR_RCP_ and with CAD (all *P* for nonlinear > 0.05; Figures 1B, D). With increasing HR, the risk of CAD is reduced sharply. Nonlinear relationships of HR_max_ and HR_Rec60s_ with CAD were observed (all *P* for nonlinear < 0.05; Figures 1F, H).

## 4 Discussion

This present study aimed to investigate the association of HR_AT_, HR_RCP_, HR_max_, and HR_Rec60s_ assessed by CPET with CAD. Analyzing a robust sample of 705 participants—322 without CAD and 383 with CAD—we mainly found that each additional beat per minute in HR_AT_ was associated with a 2.8% lower adjusted risk of CAD, and each additional beat per minute in HR_RCP_ was linked to a 2.6% lower adjusted risk of CAD in the general population. Referring to participants with HR_AT_ < 96 bpm, the risk of CAD of the participants with HR_AT_ ranged from 96 to 110 bpm and ≥110 bpm lower 38.2% and 63.2%, respectively. Referring to participants with HR_RCP_ < 111 bpm, the risk of CAD of the participants with HR_RCP_ ranged from 111 to 127 bpm and ≥127 bpm lower 60.6% and 66.4%, respectively. Further subgroup analysis showed significant interactions between HR_AT_ and sex, hypertension and lung cancer; HR_RCP_ and sex and hypertension in relation to CAD. The Dose-Response Analysis revealed with increasing HR_AT_ and HR_RCP_, the risk of CAD is reduced sharply. These results are suggestive of a good association between HR_AT_ and HR_RCP_ with CAD. Thus, it is necessary to fully exploit the potential clinic diagnostic value of HR_AT_ and HR_RCP_.

Around the world, the prevalence of CAD has increased dramatically due to an aging population, unhealthy lifestyles, and environmental changes following decades of rapid economic development. According to the Report on Cardiovascular Health and Diseases in China 2022 (21): An Updated Summary, the number of current CAD patients in China is estimated to be 11.39 million. Early diagnosis and recognition of CAD is essential. Thoroughly exploring the clinical diagnostic value of CPET is required, as it is a more sensitive and comprehensive method of screening for CAD than resting electrocardiogram (ECG) and ECG-only cardiac stress testing in adults with suspected CAD, especially asymptomatic people (22). The gas analysis can detect myocardial ischemia with reduced pulse volume and cardiac output during exercise before ST-segment changes or chest pain develops (23).

The evidence base for CPET screening and diagnosing CAD has grown exponentially over the past few decades. The European Association for Cardiovascular Prevention and Rehabilitation (EACPR) and American Heart Association (AHA) recommended a diagnostic stratification chart for patients with suspected myocardial ischemia, applying primary CPET variables such as O_2_ pulse trajectory, per cent-predicted Peak oxygen uptake (VO_2peak_), and ΔVO_2_/ΔWR trajectory (24). In this chart, the progressive variables are indicative of poorer aerobic fitness and possibly increased CAD severity. Further, existing studies suggested respiratory equivalent during anaerobic threshold (VE/VCO_2_) (25, 26), VO_2peak_ (27), time to reach the anaerobic threshold (TAT) (28), VO_2_/HR (29), the ratio of the increase in VO_2_ to the increase in work rate (ΔVO_2_/ΔWR) (22, 23) can be abnormal for CAD patients thus them are also significant parameters supporting diagnosis of CAD. However, for most of these CPET variables, a prerequisite for their accuracy and suggestive value is that the patients reach peak exercise at or near maximum effort or have a RER greater than 1.05. In the clinical process, it was hard for CAD patients, especially those with severe symptoms or long-term no exercise, to perform the near-maximal effort in the cardiopulmonary exercise test. Approximately 4% to 22% of patients with cardiovascular disease fail to reach peak effort due to premature interruption of exercise testing for some motivational or emotional (anxiety) reason or medical reasons assessed by the supervisor (30).

During exercise with progressively increasing workload, ventilation follows three distinct domains regulated respectively by oxygen uptake, carbon dioxide production, and unbuffered acidosis. The entire progressive exercise is therefore divided into three domains in order: from beginning to AT, between AT and RCP, and from RCP to the end (31). AT is a submaximal index of exercise capacity, which signifies a metabolic transition toward increased glycolysis and raised lactate with an associated metabolic acidosis (32). The RCP is a point that marks the onset of hyperventilation during incremental exercise, which forms the boundary between the heavy and severe exercise intensity domains (33). Very few studies have reported the relationship between AT, RCP, and cardiovascular disease, especially CAD. Nakade et al. demonstrated that the duration between the RCP and AT (RCP-AT time) can predict the severity of cardiac disorders and prognosis in patients with heart failure with reduced fraction ejection (34). Alberto et al. found RCP-AT time significantly predicts CAD in patients with anginal chest pain and Left bundle branch block. Our study focused on the HR at AT and RCP in CAD patients for the first time (28).

The acute HR response to exercise, HR increase during exercise, and HR recovery after exercise provide unique insights into cardiac physiology compared to resting HR and can therefore be used to gain more information about cardiac function (35, 36). An impaired HR response to exercise (i.e., chronotropic incompetence, CI) has been shown to be predictive of all-cause mortality and risk of incident CAD, even after accounting for age, physical fitness, and standard cardiovascular risk factors (37). CI is commonly considered when (1) HR_max_ during exercise < 85% of the maximal age-predicted heart rate; or (2) failure to attain 80% of heart rate reserve (38). However, it is vital to consider the patient's level of effort and the reason for terminating the exercise test before diagnosing CI (39). That means the conclusion of CI requires that the patient perform near-maximal effort in CPET. For CAD patients who find it hard to reach peak effort, how do we properly find “poor HR response to exercise”? Our main finding of association between CAD and HR_AT_ or HR_RCP_ perhaps provide a potential clinic diagnostic value to find impaired chronotropic response upon heavy not severe intensity exercise.

The HR at any moment reflects the dynamic balance between sympathetic and parasympathetic nerves in the autonomic nervous system. Unlike resting HR, exercise HR is also largely influenced by cardiorespiratory fitness (40). The gradually increasing HR is the most significant contributor to the ability to sustain aerobic exercise. An intact HR response is essential to closely match a patient's cardiac output to the metabolic demands of exercise. Not only the inability to achieve maximal HR, submaximal HR insufficiency, or HR instability during exertion are all signs of an impaired chronotropic response (39). These complaints are relatively common in CAD, sick sinus syndrome, atrioventricular block, heart failure, and so on. Based on our analysis, the lower HR_AT_, HR_RCP_, and HR_max_ are signs of impaired chronotropic response in CAD. HR_AT_ and HR_RCP_ are potentially good indicators of impaired chronotropic response without considering maximal effort. In addition, the underlying mechanisms for CI in CAD and other cardiovascular disorders are incompletely understood. Referring to the mechanism of CI, we assumed that lower HR_AT_ and HR_RCP_ for CAD are related to autonomic nervous system dysfunction. These results are suggestive of a good association between HR_AT_, HR_RCP_, and CAD. Based on our analysis, the lower HR_AT_, HR_RCP_, and HR_max_ are signs of impaired chronotropic response in CAD. HR_AT_ and HR_RCP_ are potentially good indicators of impaired chronotropic response without considering maximal effort.

The study has potential limitation. First, the patients included in this study were all from Beijing Hospital and only represented a single-center study. The sample size of patients included was limited. Further confirmation clinic trials involving larger sample sizes and multiple centers are necessary. Second, this study was cross-sectional and does not allow for causal inferences; a longitudinal study is needed before forming any causal links. Third, the degree of stenosis of the coronary arteries in the included population should be graded to assess the association between HR and CAD further, but limited due to that not every participant had a result of invasive coronary angiography or coronary computer tomography angiography. Fourth, the diagnostic value of HR_AT_ and HR_RCP_ for suspected CAD is inappropriate for patients who cannot exercise and/or augment the HR response (advanced CAD, autonomic dysfunction, and HR-limiting medications causing CI).

## 5 Conclusion

These results are suggestive of a good association between HR_AT_, HR_RCP_, and CAD. The lower HR_AT_, HR_RCP_, and HR_max_ are signs of poor HR response to exercise in CAD. HR_AT_ and HR_RCP_ are potentially good indicators of poor HR response to exercise without considering maximal effort.

## Data Availability

The original contributions presented in the study are included in the article/Supplementary Material, further inquiries can be directed to the corresponding authors.
