# Supplementary material for: The association of coronary artery disease with heart rate at anaerobic threshold and respiratory compensatory point
Source: Front Cardiovasc Med. 2024 Oct 2;11:1442857. doi: 10.3389/fcvm.2024.1442857 (PMC11479955; doi:10.3389/fcvm.2024.1442857)
Supplement: Supplementary file 1 [file Table1.pdf]

Supplementary Material

1 Supplementary Tables

Supplementary Table 1. Baseline characteristics of 705 participants grouped by HR_{AT}.

	HR_{AT}<96 (n=229)	96≤HR_{AT}<110 (n=236)	HR_{AT}≥110 (n=240)	*P*
CHD (%)	162 (70.74)	131 (55.51)	90 (37.50)	<0.0001
Sex (%)				0.0011
Female	78 (34.06)	97 (41.10)	122 (50.83)	
Male	151 (65.94)	139 (58.90)	118 (49.17)	
Age (year), mean (SD)	63.227 (8.925)	59.729 (10.833)	55.438 (12.806)	<0.0001
BMI, median (IQR)	25.535 (23.191, 27.513)	24.974 (23.174, 27.336)	24.340 (22.309, 27.226)	0.017
BMI (%)				0.0233
Normal	105 (45.85)	117 (49.58)	131 (54.58)	
Overweight	123 (53.71)	116 (49.15)	101 (42.08)	
Low	1 (0.44)	3 (1.27)	8 (3.33)	
Hypertension (%)	179 (78.17)	159 (67.37)	135 (56.25)	<0.0001
DM (%)	70 (30.57)	67 (28.39)	68 (28.33)	0.8331
Hyperlipidemia (%)	169 (73.80)	159 (67.37)	130 (54.17)	<0.0001
CKD (%)	16 (6.99)	8 (3.39)	11 (4.58)	0.1921
Lung caner (%)	41 (17.90)	51 (21.61)	53 (22.08)	0.475
Stroke (%)	29 (12.66)	22 (9.32)	14 (5.83)	0.0381
OSA (%)	14 (6.11)	11 (4.66)	13 (5.42)	0.7861
COPD (%)	9 (3.93)	5 (2.12)	7 (2.92)	0.5157
**Serum indexes**				

NT-proBNP (pg/ml), mean (SD)	275.176 (553.972)	229.899 (885.720)	247.472 (638.021)	0.7856
BNP (pg/ml), mean (SD)	88.279 (134.071)	68.699 (83.735)	70.685 (75.459)	0.0707
TC (mmol/L), mean (SD)	4.086 (0.913)	4.430 (1.004)	4.544 (0.937)	<0.0001
TG (mmol/L), mean (SD)	1.621 (1.218)	1.537 (1.272)	1.513 (0.928)	0.5716
HDL-C (mmol/L), mean (SD)	1.140 (0.296)	1.189 (0.281)	1.231 (0.306)	0.0039
LDL-C (mmol/L), mean (SD)	2.336 (0.809)	2.622 (0.888)	2.752 (0.813)	<0.0001
**Resting**				
VO_2 (L/min), median (IQR)	321.469 (267.000, 389.000)	316.000 (262.750, 380.500)	310.500 (255.000, 373.000)	0.6885
VO_2/kg (ml/kg/min), median (IQR)	4.400 (3.800, 5.200)	4.500 (3.800, 5.400)	4.600 (3.900, 5.500)	0.2694
HR (bpm), median (IQR)	68.000 (63.000, 75.000)	77.000 (71.750, 83.000)	86.000 (80.000, 94.250)	<0.0001
VO_2/HR (ml/beat), median (IQR)	4.800 (3.900, 5.700)	4.200 (3.500, 5.000)	3.600 (3.000, 4.325)	<0.0001
**AT**				
VO_2 (L/min), median (IQR)	741.000 (588.000, 865.000)	841.000 (691.608, 1049.000)	988.000 (763.500, 1219.250)	<0.0001
VO_2/kg (ml/kg/min), median (IQR)	10.100 (8.800, 11.600)	12.100 (10.400, 14.025)	14.050 (12.000, 17.325)	<0.0001
HR (bpm), median (IQR)	88.000 (83.000, 92.000)	103.000 (100.000, 106.000)	118.000 (114.000, 127.500)	<0.0001
VO_2/HR (ml/beat), median (IQR)	8.400 (6.700, 10.200)	8.250 (6.800, 10.125)	8.100 (6.300, 10.125)	0.4002
$\Delta VO_2/\Delta WR$ (ml/min/Watt), median (IQR)	9.630 (7.860, 11.180)	9.855 (8.392, 11.288)	9.771 (8.393, 11.750)	0.5628
**Peak**				
VO_2 (L/min), median (IQR)	1212.000 (937.000, 1461.000)	1321.000 (1058.250, 1623.250)	1414.500 (1117.653, 1730.000)	<0.0001
VO_2/kg (ml/kg/min), median (IQR)	16.500 (14.300, 19.400)	18.900 (15.800, 22.425)	21.050 (17.400, 24.725)	<0.0001
HR (bpm), median (IQR)	113.000 (101.000, 121.000)	131.000 (121.000, 142.242)	148.000 (137.000, 160.000)	<0.0001
VO_2/HR (ml/beat), median (IQR)	10.700 (8.700, 12.800)	10.000 (8.100, 12.100)	9.450 (7.700, 12.025)	0.0024

$\Delta VO_2/\Delta WR$ (ml/min/Watt), median (IQR)	9.820 (8.560, 11.180)	9.773 (8.640, 10.880)	9.595 (7.700, 10.990)	0.3865
**RCP**				
VO_2 (L/min), median (IQR)	967.000 (795.000, 1201.000)	1082.455 (880.750, 1367.750)	1218.500 (946.000, 1507.000)	<0.0001
VO_2/kg (ml/kg/min), median (IQR)	13.400 (11.700, 16.000)	15.750 (13.593, 18.300)	17.900 (14.700, 21.600)	<0.0001
HR (bpm), median (IQR)	100.000 (96.835, 108.000)	118.000 (113.000, 126.073)	136.000 (129.000, 146.500)	<0.0001
VO_2/HR (ml/beat), median (IQR)	9.600 (7.900, 11.600)	9.050 (7.375, 11.400)	8.700 (7.082, 11.100)	0.0079
$\Delta VO_2/\Delta WR$ (ml/min/Watt), median (IQR)	9.240 (8.430, 10.634)	9.455 (8.245, 10.515)	9.337 (8.100, 10.662)	0.8324

Abbreviations: BMI, body mass index; DM, diabetes mellitus; CKD, chronic kidney disease; OSA, obstructive sleep apnea; COPD, chronic obstructive pulmonary disease; BNP, brain natriuretic peptide; TC, total cholesterol; TG, triglyceride; VO_2, oxygen consumption; VO_2/kg, oxygen consumption/kilogram; HR, heart rate; VO_2/HR, oxygen pulse; $\Delta VO_2/\Delta WR$, ratio of the increase in VO_2 to the increase in the work rate; AT, anaerobic threshold; RCP, respiratory compensation point; SD, standard deviation; IQR, interquartile range.

Supplementary Table 2. Baseline characteristics of 705 participants grouped by HR_{RCP}.

	**HR_{RCP}<111 (n=229)**	**111≤HR_{RCP}<127 (n=236)**	**HR_{RCP}≥127 (n=240)**	***P***
CHD (%)	165 (72.05)	123 (52.12)	95 (39.58)	<0.0001
Sex (%)				
Female	77 (33.62)	96 (40.68)	124 (51.67)	0.0003
Male	152 (66.38)	140 (59.32)	116 (48.33)	
Age (year), mean (SD)	63.865 (9.042)	59.581 (11.386)	54.975 (11.875)	<0.0001
BMI, median (IQR)	25.391 (23.140, 27.732)	25.071 (23.133, 27.371)	24.221 (22.403, 26.685)	0.004
BMI (%)				
Normal	104 (45.41)	112 (47.46)	137 (57.08)	0.0358
Overweight	123 (53.71)	120 (50.85)	97 (40.42)	
Low	2 (0.87)	4 (1.69)	6 (2.50)	
Hypertension (%)	181 (79.04)	152 (64.41)	140 (58.33)	<0.0002
DM (%)	72 (31.44)	70 (29.66)	63 (26.25)	0.4517
Hyperlipidemia (%)	163 (71.18)	163 (69.07)	132 (55.00)	0.0003
CKD (%)	16 (6.99)	6 (2.54)	13 (5.42)	0.0811
Lung caner (%)	50 (21.83)	44 (18.64)	51 (21.25)	0.6611
Stroke (%)	28 (12.23)	20 (8.47)	17 (7.08)	0.1395
OSA (%)	15 (6.55)	14 (5.93)	9 (3.75)	0.3667
COPD (%)	12 (5.24)	6 (2.54)	3 (1.25)	0.0353
**Serum indexes**				
NT-proBNP (pg/ml), mean (SD)	352.796 (1028.593)	202.093 (434.400)	200.752 (512.381)	0.0286
BNP (pg/ml), mean (SD)	92.708 (140.312)	69.796 (77.029)	65.381 (70.193)	0.0072
TC (mmol/L), mean (SD)	4.084 (0.966)	4.440 (0.952)	4.536 (0.940)	<0.0001

TG (mmol/L), mean (SD)	1.564 (1.028)	1.667 (1.518)	1.440 (0.757)	0.0973
HDL-C (mmol/L), mean (SD)	1.144 (0.301)	1.167 (0.282)	1.248 (0.297)	0.0003
LDL-C (mmol/L), mean (SD)	2.345 (0.851)	2.626 (0.845)	2.739 (0.822)	<0.0001
**Resting**				
VO_2 (L/min), median (IQR)	325.000 (273.000, 391.000)	315.035 (260.750, 389.000)	310.500 (256.500, 370.000)	0.3339
VO_2/kg (ml/kg/min), median (IQR)	4.500 (3.900, 5.200)	4.400 (3.800, 5.400)	4.600 (3.900, 5.500)	0.4116
HR (bpm), median (IQR)	70.000 (64.000, 76.000)	78.000 (71.000, 85.000)	84.000 (77.000, 92.250)	<0.0001
VO_2/HR (ml/beat), median (IQR)	4.700 (3.800, 5.600)	4.200 (3.400, 4.900)	3.700 (3.000, 4.400)	<0.0001
**AT**				
VO_2 (L/min), median (IQR)	738.000 (591.000, 856.000)	76.000 (688.000, 1090.500)	954.000 (752.500, 1191.000)	<0.0001
VO_2/kg (ml/kg/min), median (IQR)	10.100 (8.800, 11.700)	12.300 (10.200, 14.925)	13.600 (11.675, 17.200)	<0.0001
HR (bpm), median (IQR)	88.000 (83.000, 93.000)	104.000 (99.000, 109.000)	118.000 (111.000, 127.500)	<0.0001
VO_2/HR (ml/beat), median (IQR)	8.100 (6.700, 9.900)	8.600 (6.675, 10.500)	8.100 (6.436, 10.125)	0.2161
$\Delta VO_2/\Delta WR$ (ml/min/Watt), median (IQR)	9.550 (7.810, 11.180)	10.065 (8.630, 11.810)	9.735 (8.409, 11.502)	0.0869
**Peak**				
VO_2 (L/min), median (IQR)	1127.000 (911.000, 1391.000)	44.000 (1076.500, 1625.500)	1464.500 (1161.000, 1767.500)	<0.0001
VO_2/kg (ml/kg/min), median (IQR)	15.800 (13.700, 18.600)	19.000 (16.250, 22.700)	21.350 (18.075, 25.550)	<0.0001
HR (bpm), median (IQR)	110.000 (102.000, 118.000)	130.000 (122.750, 136.000)	151.000 (144.000, 162.000)	<0.0001
VO_2/HR (ml/beat), median (IQR)	10.500 (8.366, 12.600)	10.300 (8.300, 12.400)	9.450 (7.700, 11.825)	0.005
$\Delta VO_2/\Delta WR$ (ml/min/Watt), median (IQR)	9.700 (8.400, 11.330)	9.970 (8.850, 11.177)	9.495 (7.825, 10.578)	0.0032
**RCP**				
VO_2 (L/min), median (IQR)	938.000 (755.000, 1136.070)	136.500 (880.750, 1398.000)	1233.000 (981.750, 1516.500)	<0.0001
VO_2/kg (ml/kg/min), median (IQR)	13.000 (11.400, 14.900)	15.900 (13.600, 18.701)	18.400 (15.150, 21.800)	<0.0001

HR (bpm), median (IQR)	100.000 (96.627, 106.000)	118.000 (115.000, 123.000)	139.000 (133.000, 148.000)	<0.0001
VO_2/HR (ml/beat), median (IQR)	9.200 (7.800, 11.400)	9.610 (7.500, 11.600)	8.700 (7.200, 11.025)	0.0236
$\Delta VO_2/\Delta WR$ (ml/min/Watt), median (IQR)	9.240 (8.260, 10.716)	9.655 (8.545, 10.787)	9.320 (8.058, 10.330)	0.1044

Abbreviations: BMI, body mass index; DM, diabetes mellitus; CKD, chronic kidney disease; OSA, obstructive sleep apnea; COPD, chronic obstructive pulmonary disease; BNP, brain natriuretic peptide; TC, total cholesterol; TG, triglyceride; VO_2, oxygen consumption; VO_2/kg, oxygen consumption/kilogram; HR, heart rate; VO_2/HR, oxygen pulse; $\Delta VO_2/\Delta WR$, ratio of the increase in VO_2 to the increase in the work rate; AT, anaerobic threshold; RCP, respiratory compensation point; SD, standard deviation; IQR, interquartile range.

Supplementary Table 3. Baseline characteristics of 705 participants grouped by HR_{max}.

	**HR_{max}<121 (n=225)**	**121≤HR_{max}<142 (n=239)**	**HR_{max}≥142 (n=241)**	***P***
CHD (%)	159 (70.67)	131 (54.81)	93 (38.59)	<0.0001
Sex (%)				0.0074
Female	85 (37.78)	91 (38.08)	121 (50.21)	
Male	140 (62.22)	148 (61.92)	120 (49.79)	
Age (year), mean (SD)	63.618 (9.665)	60.803 (10.249)	54.083 (12.053)	<0.0001
BMI, median (IQR)	25.153 (23.030, 27.539)	25.102 (23.437, 27.451)	24.337 (22.309, 26.730)	0.0121
BMI (%)				0.1486
Normal	104 (46.22)	115 (48.12)	134 (55.60)	
Overweight	118 (52.44)	121 (50.63)	101 (41.91)	
Low	3 (1.33)	3 (1.26)	6 (2.49)	
Hypertension (%)	176 (78.22)	162 (67.78)	135 (56.02)	<0.0001
DM (%)	74 (32.89)	68 (28.45)	63 (26.14)	0.2674
Hyperlipidemia (%)	158 (70.22)	165 (69.04)	135 (56.02)	0.0015
CKD (%)	16 (7.11)	9 (3.77)	10 (4.15)	0.1955
Lung caner (%)	53 (23.56)	49 (20.50)	43 (17.84)	0.3126
Stroke (%)	29 (12.89)	20 (8.37)	16 (6.64)	0.0566
OSA (%)	14 (6.22)	15 (6.28)	9 (3.73)	0.3736
COPD (%)	10 (4.44)	8 (3.35)	3 (1.24)	0.1169
**Serum indexes**				
NT-proBNP (pg/ml), mean (SD)	357.461 (1037.100)	216.917 (460.183)	184.202 (485.739)	0.0199
BNP (pg/ml), mean (SD)	92.270 (140.980)	74.608 (87.870)	61.416 (56.263)	0.0041
TC (mmol/L), mean (SD)	4.108 (0.924)	4.382 (0.973)	4.564 (0.962)	<0.0001

TG (mmol/L), mean (SD)	1.561 (0.999)	1.629 (1.455)	1.479 (0.902)	0.3588
HDL-C (mmol/L), mean (SD)	1.169 (0.305)	1.160 (0.289)	1.231 (0.291)	0.0186
LDL-C (mmol/L), mean (SD)	2.346 (0.805)	2.581 (0.842)	2.778 (0.861)	<0.0001
**Resting**				
VO_2 (L/min), median (IQR)	315.000 (271.000, 382.000)	319.000 (259.500, 388.000)	314.000 (259.000, 375.000)	0.7724
VO_2/kg (ml/kg/min), median (IQR)	4.600 (3.900, 5.300)	4.400 (3.800, 5.300)	4.600 (3.900, 5.600)	0.1913
HR (bpm), median (IQR)	71.000 (64.000, 78.000)	76.000 (69.000, 84.000)	83.000 (77.000, 91.000)	<0.0001
VO_2/HR (ml/beat), median (IQR)	4.600 (3.700, 5.500)	4.300 (3.400, 5.137)	3.800 (3.100, 4.500)	<0.0001
**AT**				
VO_2 (L/min), median (IQR)	745.000 (595.000, 878.000)	862.000 (688.500, 1074.000)	927.000 (741.000, 1188.000)	<0.0001
VO_2/kg (ml/kg/min), median (IQR)	10.300 (9.000, 12.300)	12.200 (10.100, 14.300)	13.400 (11.400, 17.100)	<0.0001
HR (bpm), median (IQR)	90.000 (84.895, 96.000)	104.000 (95.000, 110.500)	116.000 (108.000, 127.000)	<0.0001
VO_2/HR (ml/beat), median (IQR)	8.100 (6.600, 9.900)	8.600 (6.750, 10.500)	7.900 (6.448, 10.200)	0.2195
$\Delta VO_2/\Delta WR$ (ml/min/Watt), median (IQR)	9.550 (7.810, 11.060)	10.140 (8.745, 12.070)	9.730 (8.220, 11.180)	0.0235
**Peak**				
VO_2 (L/min), median (IQR)	1108.000 (883.000, 1368.000)	1360.000 (1097.000, 1614.000)	1476.000 (1220.000, 1854.000)	<0.0001
VO_2/kg (ml/kg/min), median (IQR)	15.600 (13.700, 18.400)	18.500 (16.300, 21.850)	22.000 (18.700, 26.100)	<0.0001
HR (bpm), median (IQR)	110.000 (101.000, 116.000)	130.000 (125.000, 134.000)	153.000 (146.000, 162.000)	<0.0001
VO_2/HR (ml/beat), median (IQR)	10.400 (8.200, 12.700)	10.500 (8.500, 12.150)	9.300 (8.000, 12.100)	0.0337
$\Delta VO_2/\Delta WR$ (ml/min/Watt), median (IQR)	9.910 (8.640, 11.460)	9.860 (8.755, 11.190)	9.440 (7.770, 10.450)	0.0001
**RCP**				
VO_2 (L/min), median (IQR)	931.000 (746.000, 1151.000)	1124.000 (891.000, 1353.000)	1225.000 (979.000, 1544.000)	<0.0001

VO_2/kg (ml/kg/min), median (IQR)	13.200 (11.600, 15.500)	15.700 (13.300, 18.200)	18.100 (14.900, 21.800)	<0.0001
HR (bpm), median (IQR)	101.000 (96.627, 109.000)	118.000 (113.000, 125.780)	137.380 (130.000, 148.000)	<0.0001
VO_2/HR (ml/beat), median (IQR)	9.200 (7.400, 11.300)	9.600 (7.850, 11.350)	8.700 (7.200, 11.200)	0.1412
$\Delta VO_2/\Delta WR$ (ml/min/Watt), median (IQR)	9.340 (8.426, 10.720)	9.510 (8.320, 10.795)	9.310 (7.910, 10.280)	0.047

Abbreviations: BMI, body mass index; DM, diabetes mellitus; CKD, chronic kidney disease; OSA, obstructive sleep apnea; COPD, chronic obstructive pulmonary disease; BNP, brain natriuretic peptide; TC, total cholesterol; TG, triglyceride; VO_2, oxygen consumption; VO_2/kg, oxygen consumption/kilogram; HR, heart rate; VO_2/HR, oxygen pulse; $\Delta VO_2/\Delta WR$, ratio of the increase in VO_2 to the increase in the work rate; AT, anaerobic threshold; RCP, respiratory compensation point; SD, standard deviation; IQR, interquartile range.

Supplementary Table 4. Baseline characteristics of 705 participants grouped by HR_{Rec60s}.

	HR_{Rec60s}<105 (n=231)	105≤HR_{Rec60s}<123 (n=234)	HR_{Rec60s}≤123 (n=240)	*P*
CHD (%)	163 (70.56)	117 (50.00)	103 (42.92)	<0.0001
Sex (%)				0.098
Female	94 (40.69)	89 (38.03)	114 (47.50)	
Male	137 (59.31)	145 (61.97)	126 (52.50)	
Age (year), mean (SD)	63.597 (9.201)	59.585 (10.985)	55.192 (12.302)	<0.0001
BMI, median (IQR)	25.161 (23.233, 27.316)	25.055 (23.147, 27.734)	24.337 (22.309, 26.715)	0.0068
BMI (%)				0.1032
Normal	107 (46.32)	112 (47.86)	134 (55.83)	
Overweight	122 (52.81)	118 (50.43)	100 (41.67)	
Low	2 (0.87)	4 (1.71)	6 (2.50)	
Hypertension (%)	179 (77.49)	154 (65.81)	140 (58.33)	<0.0001
DM (%)	72 (31.17)	64 (27.35)	69 (28.75)	0.6568
Hyperlipidemia (%)	163 (70.56)	158 (67.52)	137 (57.08)	0.0055
CKD (%)	14 (6.06)	9 (3.85)	12 (5.00)	0.5463
Lung caner (%)	55 (23.81)	49 (20.94)	41 (17.08)	0.1931
Stroke (%)	30 (12.99)	19 (8.12)	16 (6.67)	0.0468
OSA (%)	14 (6.06)	13 (5.56)	11 (4.58)	0.7701
COPD (%)	11 (4.76)	6 (2.56)	4 (1.67)	0.1281
**Serum indexes**				
NT-proBNP (pg/ml), mean (SD)	313.685 (568.385)	238.382 (957.142)	201.759 (511.673)	0.2173
BNP (pg/ml), mean (SD)	86.330 (131.796)	71.372 (95.031)	69.792 (65.409)	0.148
TC (mmol/L), mean (SD)	4.132 (0.949)	4.417 (0.936)	4.516 (0.989)	<0.0001

TG (mmol/L), mean (SD)	1.518 (0.929)	1.695 (1.542)	1.458 (0.832)	0.0653
HDL-C (mmol/L), mean (SD)	1.161 (0.306)	1.167 (0.274)	1.231 (0.303)	0.0173
LDL-C (mmol/L), mean (SD)	2.386 (0.825)	2.595 (0.842)	2.733 (0.862)	<0.0001
**Resting**				
VO_2 (L/min), median (IQR)	313.000 (267.000, 381.500)	324.000 (262.250, 382.750)	309.500 (258.500, 386.500)	0.881
VO_2/kg (ml/kg/min), median (IQR)	4.500 (3.800, 5.300)	4.500 (3.900, 5.200)	4.700 (3.900, 5.525)	0.253
HR (bpm), median (IQR)	69.000 (64.000, 76.000)	77.000 (71.000, 83.750)	85.000 (78.000, 93.000)	<0.0001
VO_2/HR (ml/beat), median (IQR)	4.700 (3.800, 5.500)	4.200 (3.500, 4.900)	3.700 (3.000, 4.600)	<0.0001
**AT**				
VO_2 (L/min), median (IQR)	752.000 (594.500, 890.500)	862.500 (707.000, 1064.000)	949.500 (726.750, 1180.500)	<0.0001
VO_2/kg (ml/kg/min), median (IQR)	10.400 (9.000, 12.400)	12.200 (10.300, 14.175)	13.450 (11.200, 17.225)	<0.0001
HR (bpm), median (IQR)	90.000 (84.000, 96.000)	104.000 (97.000, 109.997)	117.000 (108.000, 127.500)	<0.0001
VO_2/HR (ml/beat), median (IQR)	8.100 (6.631, 10.000)	8.550 (6.910, 10.375)	8.050 (6.300, 10.200)	0.172
$\Delta VO_2/\Delta WR$ (ml/min/Watt), median (IQR)	9.690 (7.995, 11.150)	9.695 (8.555, 11.675)	9.735 (8.155, 11.695)	0.6619
**Peak**				
VO_2 (L/min), median (IQR)	1112.000 (884.500, 1374.500)	1350.500 (1123.000, 1620.250)	1464.500 (1187.750, 1819.500)	<0.0001
VO_2/kg (ml/kg/min), median (IQR)	15.800 (13.650, 18.550)	19.000 (16.125, 22.075)	21.600 (17.975, 26.025)	<0.0001
HR (bpm), median (IQR)	110.000 (102.000, 118.000)	131.000 (125.000, 136.000)	151.000 (144.000, 162.000)	<0.0001
VO_2/HR (ml/beat), median (IQR)	10.000 (8.100, 12.650)	10.600 (8.462, 12.200)	9.550 (7.716, 12.125)	0.0547
$\Delta VO_2/\Delta WR$ (ml/min/Watt), median (IQR)	9.950 (8.655, 11.365)	9.835 (8.728, 11.012)	9.420 (7.695, 10.640)	0.0034
**RCP**				
VO_2 (L/min), median (IQR)	938.000 (743.500, 1165.500)	1121.581 (909.500, 1383.500)	1229.500 (958.250, 1537.250)	<0.0001

VO_2/kg (ml/kg/min), median (IQR)	13.300 (11.507, 15.750)	15.850 (13.525, 18.175)	18.095 (14.675, 21.806)	<0.0001
HR (bpm), median (IQR)	101.000 (96.948, 109.000)	120.000 (113.074, 126.000)	137.000 (130.000, 148.000)	<0.0001
VO_2/HR (ml/beat), median (IQR)	9.200 (7.493, 11.306)	9.550 (7.625, 11.400)	8.700 (7.175, 11.200)	0.0925
$\Delta VO_2/\Delta WR$ (ml/min/Watt), median (IQR)	9.400 (8.428, 10.713)	9.444 (8.442, 10.641)	9.290 (7.787, 10.400)	0.1758

Abbreviations: BMI, body mass index; DM, diabetes mellitus; CKD, chronic kidney disease; OSA, obstructive sleep apnea; COPD, chronic obstructive pulmonary disease; BNP, brain natriuretic peptide; TC, total cholesterol; TG, triglyceride; VO_2, oxygen consumption; VO_2/kg, oxygen consumption/kilogram; HR, heart rate; VO_2/HR, oxygen pulse; $\Delta VO_2/\Delta WR$, ratio of the increase in VO_2 to the increase in the work rate; AT, anaerobic threshold; RCP, respiratory compensation point; Rec60s, post-exercise after 60 seconds; SD, standard deviation; IQR, interquartile range.
